# Simulated driving behavior over the adult age span

**DOI:** 10.3389/fnagi.2025.1496224

**Published:** 2025-02-19

**Authors:** Felix Menze, Nathan W. Churchill, Tom A. Schweizer, Simon J. Graham

**Affiliations:** ^1^Department of Medical Biophysics, University of Toronto, Toronto, ON, Canada; ^2^Sunnybrook Health Sciences Centre, Sunnybrook Research Institute, Toronto, ON, Canada; ^3^Keenan Research Centre for Biomedical Science, Li Ka Shing Knowledge Institute, St. Michael’s Hospital, Toronto, ON, Canada; ^4^Brain Health and Wellness Research Program, St. Michael’s Hospital, Unity Health Toronto, Toronto, ON, Canada; ^5^Department of Physics, Toronto Metropolitan University, Toronto, ON, Canada; ^6^Faculty of Medicine (Neurosurgery), University of Toronto, Toronto, ON, Canada

**Keywords:** driving, distracted driving, driving simulation, aging, maturation, cognition, driving safety

## Abstract

**Background:**

Motor vehicle accidents remain a leading cause of accidental death worldwide. Death and injury rates are particularly high for both young inexperienced drivers and elderly drivers. Understanding the behavioral changes that are associated with maturation and aging could inform assessments of driving performance and lead to new measures identifying at-risk drivers. To shed further light on such effects, this study aims to characterize simulated driving behavior across and within age groups using a large driving simulation dataset.

**Methods:**

The analyzed dataset consisted of 112 participants [47/112 (42%) female] between the ages of 17 and 85 (average ± standard deviation: 54 ± 22 years). Participants performed navigation in scenarios modeled after the standard licensing test of Ontario, Canada, which included a series of turns at intersections with different levels of complexity (e.g., involving oncoming traffic or pedestrians) and levels of distraction (requiring auditory responses to common-knowledge questions). Behavioral metrics were defined and investigated not only for the full completion of each task but also based on common subtasks (e.g., braking at an intersection), which were then compared across and within age groups (young, middle-aged, old).

**Results:**

Overall, young adults behaved similarly to middle-aged adults for basic tasks but showed differences during traffic navigation subtasks when distracted, such as starting to decelerate significantly later when approaching intersections. Old drivers, on the other hand, drove at lower average speed, stopped earlier at intersections, and left increased distances to pedestrians, but required significantly more time to complete the driving tasks.

**Conclusion:**

With rich detail arising from intra-task quantification, the results were consistent with and additive to previous literature showcasing that compared to middle-aged adults, young adults showed performance suggestive of riskier driving behavior, and old adults showed performance suggestive of caution consequent to declining driving ability. In particular, the intra-task quantification revealed that the driving of young adults was more impacted by the presence of distraction (e.g., delayed decelerating), whereas old adults prioritized safe driving (e.g., correctly braking at intersections) over responding to distractions. The study may be used as motivation for future studies of driving safety and accident prevention, and informed assessment of governmental regulations.

## 1 Introduction

Motor vehicle accidents cause over a million fatalities each year and remain one of the leading causes of accidental death worldwide (WHO, 2021). The age of a driver is an important risk factor in this context, with an increased rate of fatal crashes - as well as higher driver responsibility for accidents - seen among both young adults and seniors (Tefft et al., 2013). Young adult drivers cause the highest overall number of deaths, mostly consisting of pedestrians and cyclists rather than the driver themselves (NSC, 2021). Accidents are mostly caused either by detrimental circumstances such as drunk driving and smartphone usage, or by poor driving performance such as lapses in judgment or loss of control (Arafa et al., 2020; Ventsislavova et al., 2021). Risky and careless driving behavior has been reported to occur predominantly among drivers under the age of 26, likely due to the last stages of brain development being incomplete in young adults, coupled with their relatively new driving skills (Begg and Langley, 2001; Walshe et al., 2017). Driving performance subsequently increases with years of experience, plateaus in middle age, and then starts to decrease later in life as the effects of aging start to manifest (Karthaus and Falkenstein, 2016; Pope et al., 2017). For older drivers, crashes often result in severe injury or death and although there are fewer drivers above the age of 65 than in any other age group, the fatality rate for drivers themselves during crash involvement is at its peak in this demographic (NSC, 2021).

Assessment of the ability to drive safely is essential to maintain road safety. The procedures to obtain a driver’s license through written paper tests and road tests remain critically important front-line measures, which are usually completed in young adulthood and largely support safe driving until middle age. However, as driving abilities start to decrease in the elderly (Falkenstein et al., 2020), how best to maintain road safety remains a much more open question associated with this demographic. An important early step to address this concern involves investigating the age-dependent effects on driving ability, and is the focus of the present work. On-road assessments are impractical to conduct in a research context involving a large study sample, although advanced monitoring technology holds promise (Seelye et al., 2017). Given the heterogeneity of driving maneuvers, which can range from simple driving on straight roads to complex turns through traffic, it is also challenging to characterize driving performance with simple behavioral tests. For greater insight, it is thus necessary to take a closer look at specific driving scenarios and maneuvers that show diminishing performance with age.

To date, studies quantifying driving behavior, toward the long-term objective of developing improved driving safety assessment tools, have been conducted at small-to-moderate sample sizes (generally below 30 volunteers). Analyses of self-reports, simulations, and field data have shown that older adults typically drive more cautiously and slowly (Pope et al., 2017; Getzmann et al., 2018; Arafa et al., 2020). Additionally, studies have demonstrated that older adults tend to maximize distances to other cars both in simulations as well as on the road (Lu and Pernía, 2000; Andrews and Westerman, 2012). Older adults also require more time to start driving again after coming to a halt in traffic and engage in other compensatory processes (Lu and Pernía, 2000; Thompson et al., 2012). The collective literature suggests that there is a clear shift in driving behavior with age, perhaps as a strategy executed in response to decreasing driving performance. Other work has shown that the ability to allocate sufficient mental resources during complex tasks varies with age, and it is negatively associated with accident rates (Thompson et al., 2012; Michaels et al., 2017). These complex age-dependent interactions make it challenging to assess driving performance outside of extensive on-road testing, although tools measuring cognition and perception during driving simulation have shown promise in recent years (Dickerson et al., 2014). Overall, there is an ongoing need to validate, replicate and extend these initial studies over much larger numbers of individuals. Larger group sizes provide the opportunity to investigate whether results from smaller studies can be replicated and provide improved detection power to evaluate variability in driving performance.

At a deeper level, probing the neural mechanisms that underpin driving performance would likely be very useful to develop improved scientific understanding of how safe and unsafe driving occurs. In this regard, non-invasive functional neuroimaging methods have an important role to play in evaluating brain activity. Pertinent literature continues to expand in this area despite the technical challenges of combining brain imaging technology with on-road driving or, more practically, simulated driving in virtual environments (Cohen et al., 2020; Kim et al., 2020; Hussain et al., 2021). Collectively, past studies in this area involving functional Near Infrared Spectroscopy (fNIRS), functional Magnetic Resonance Imaging (fMRI) and electroencephalography (EEG) suggest that driving requires both a “standard” network of mostly posterior brain regions responsible for skilled performance, and a prefrontal network which improves driving performance with experience (Harada et al., 2007; Schweizer et al., 2013; Choi et al., 2017; Karthaus et al., 2018; Li et al., 2021; Yuen et al., 2021). Scientific understanding of these networks remains in its infancy, including how they interact and how they are modulated by various factors of interest such as age, driving experience, and disease state. In this context and particularly with respect to normal aging, a critically important initial step will be to assess these modulations referenced to the appropriate baseline – middle-aged individuals – who have extensive driving experience and engage the standard network to execute the safest driving behavior in everyday life.

Furthermore, apart from data-driven analyses (Calhoun, 2018), maps of brain activity are usually generated from functional neuroimaging data by imposing analytic models in time-series analyses - toward characterizing the relationship between the neuroimaging signals and lengthy “blocks” of task performance (lasting approximately tens of seconds) during simulated driving (Schweizer et al., 2013). Thus, the activation maps are strongly influenced by how well behavior is quantified during the task execution (Brunkhorst-Kanaan et al., 2020). In the absence of better quantification, the very simplistic assumption is often made that behavior, and thus brain activity, are well-approximated as constant throughout performance of a task block. With the long-term goal of accurately assessing driving performance, nuanced characterization of driving behavior during driving tasks covering a range of cognitive complexity is necessary to enhance functional neuroimaging studies (Schretlen et al., 2003). In the context of driving, a particular driving task may consist of coming to a full stop, looking for gaps in traffic, and executing a turn, which require maintaining attention, planning ahead, and executing motor movement, respectively. As the driver inherently controls how a particular driving task is executed dynamically, quantifying how sub-components of the task are performed is likely to be highly meaningful in comparison to aggregate evaluations integrated or time-averaged over the entire task - which obscure the dynamics of mental processing. Whereas analysis of neuroimaging data is out of scope for the present study, which focuses primarily on behavioral nuances, an in-depth characterization of driving behavior would inform future studies involving fMRI, EEG, or fNIRS data.

To fill these knowledge gaps in sample size and behavioral quantification, therefore, the present study analyzes one of the largest multimodal simulated driving datasets to date, consisting of multiple driving tasks from over 150 participants across the adult age span. This dataset permits a detailed behavioral analysis of trends and differences not yet reported. As a first step, the following hypotheses are tested that: (a) intra-task behavioral components of simulated driving behavior show different age-dependent modulations in young and old adults, when each group is compared to middle-aged adults; and (b) these behavioral components show age-dependent correlations within groups of young and old adults. The work has relevance to the future development of objective metrics that can be used to assess fitness to drive, as well as to develop assistive technologies for safer driving – and relevance to an important next step: large-scale functional neuroimaging studies of simulated driving (e.g., involving this dataset), which should thus be able to depict the associated brain activity better based on enhanced behavioral characterization.

## 2 Materials and methods

### 2.1 Participants

One-hundred-and-fifty-eight participants were recruited for the study from the general population. Participants were required to be active drivers with a valid driver’s license in Ontario, Canada between the ages of 17 and 85. From this dataset, 112 participants matched the selection criteria of being part of the three age groups of interest commonly compared in driving safety literature (young adults, middle-aged adults, and old adults, omitting 41 participants who were from transitional age ranges or did not provide their age) and having completed the driving tasks without any issues such as consistently taking the wrong turns at intersections (further removing five participants from the dataset, most likely due to issues with hearing the instructions). Participants were divided into one of three cohorts. A group of young adults (17–25, *N* = 25, 68% female) was defined based on previous studies reporting significantly different driving behavior up until the age of 25 (Begg and Langley, 2001). A group of elderly adults (65–85, *N* = 64, 36% female) was defined based on the significant increases in accident rates seen in statistics as well as pertinent literature (Pope et al., 2017; NSC, 2021). Last, a group of middle-aged adults (35–55, *N* = 23, 30% female) was defined following related past literature (Arnau-Sabatés et al., 2012; Engelberg et al., 2015; Pope et al., 2017).

Study participants had no history of major neurologic or psychiatric conditions, were not taking any medication affecting blood flow or brain function, had normal or corrected-to-normal vision (MRI-compatible vision-correction goggles were provided), and additionally met MRI inclusion criteria, including not having ferro-magnetic implants and not being claustrophobic. (Note that all participants underwent fMRI, but detailed analysis of the relationship between neural activity and behavioral data falls outside the scope of the present study and will be reported elsewhere). The present study was approved by the Research Ethics Board of St. Michael’s Hospital (Unity Health Toronto) and Baycrest Hospital in Toronto, Canada, with informed, written consent provided by all participants prior to data collection. Ethics approval was required from Baycrest, as participants were recruited in part from an established database administered by this institution.

### 2.2 Driving simulation

The simulated driving tasks were implemented using STISIM Drive software (Systems Technology Inc., Hawthorne, United States). Tasks were performed while participants lay supine within the magnet bore of a 3.0 Tesla MRI system (Magnetom Skyra, Siemens Healthineers, Erlangen, DEU), to allow for simultaneous collection of behavioral and fMRI data. Using a mirror attached to the head coil, participants viewed the driving scenarios on a back-projection screen illuminated by an MRI-conditional projector (Avotec Inc., Stuart, Florida, United States). Participants made driving responses using MRI-compatible virtual reality equipment, including a steering wheel fitted with button-press capability, and foot pedals for accelerating and decelerating. Further details are reported in previous work that describes the technical specifications and high ecological validity of the driving simulator for fMRI research, as well as images of the simulator apparatus (Kan et al., 2012).

### 2.3 Data acquisition

The in-scanner driving tasks were designed to replicate the standard licensing road test of Ontario (typically 20 min in duration) and involved navigating a relatively simple simulated roadscape (Schweizer et al., 2013). Each “run” of data collection included different driving scenarios consisting of a pseudo-randomized sequence of left and right turns at controlled intersections, interleaved with segments of driving on straight roads (S). Turns at intersections included the following: making a left turn at an intersection without traffic (L), making a left turn at an intersection with oncoming traffic (LT), slowing down at a stop sign and making a right turn (R), and making a right turn at a stop sign with pedestrians crossing the road (RP). Participants wore MRI-compatible acoustic headphones and received navigation instructions using a series of automated voice recordings, similar to standard in-vehicle global positioning systems (GPS). While driving, instructions were initiated at 94 virtual metres (m) in advance of the upcoming intersection via an automated voice recording (e.g., “At the intersection, turn left”). Intersection-based driving tasks started 10 m before the instruction announcement and ended 50 m after the intersection, thus spanning a total distance of 160 m. Voice recordings also asked a series of pre-recorded general knowledge questions (e.g., “Fire trucks are green.”) as auditory distraction during a subset of tasks, which included straight driving (SA) and left turns with oncoming traffic (LTA). All participants were proficient in the English language and could accurately converse and respond to questions prior to the experiment (participants were asked after the experiment whether they were able to understand the questions during the experiments; no concerns were raised by participants, including those who were non-native speakers). Questions were asked after the appearance of the intersection, but before the participant was required to slow down. Participants had to give a “true” or “false” response using the two buttons located on the steering wheel. No prior instruction was given on whether driving or responding to the question should be prioritized, to maintain naturalistic behavior. A single run consisted of 16 unique intersections, each taking between 10 and 30 s to complete. Participants underwent two separate runs in total, each lasting approximately 10 min depending on driving speed. Across both results, this resulted in 23 repetitions for the S task; 7 for L; 7 for LT; 6 for R; 4 for RP; 6 for SA, and 7 for LTA.

Prior to data collection, participants were given approximately an hour to familiarize themselves with the driving simulator outside of the MRI system, where they practiced driving through a randomly generated sequence of the driving scenarios listed above. Participants were asked to perform all tasks to the best of their ability, while adhering to traffic laws and not exceeding the posted speed limit of 60 km/h. For the sake of avoiding distractions unless intentionally induced, the environment was made rural with minimal background scenery.

While the driving tasks were performed, the STISIM software automatically logged behavioral variables including lateral lane position, total distance traveled, vehicle speed and vehicle steering, with a sampling frequency of 30 Hz; accidents and mistakes such as crashes, off-road driving, speeding, and illegal lane crossings were also recorded. Upon task completion, these values were output in tabular format, and averages and standard deviations for the automatically generated behavioral parameters were calculated for each task using Python 3. Average speed and standard deviation in lateral lane position were of particular interest to the behavioral analysis because these two metrics corresponded to control over lateral and longitudinal movement, respectively, thus giving insight into the speeding and unsafe lane crossings habits of each driver. The vehicle steering angle was functionally dependent on the lateral lane position, distance traveled and vehicular speed, and was thus not included in the analysis. Average speed was transformed into overall duration of the task to allow for more convenient comparison with typical time-based metrics which are reported in related literature (Lu and Pernía, 2000; Karthaus et al., 2018). Because the STISIM software was unable to generate metrics correctly for uncommon events such as off-road driving or collisions with pedestrians and other vehicles, those occurrences were tracked independently from the main analysis of numerical performance metrics as continuous variables.

### 2.4 Data analysis

**FIGURE 1:**
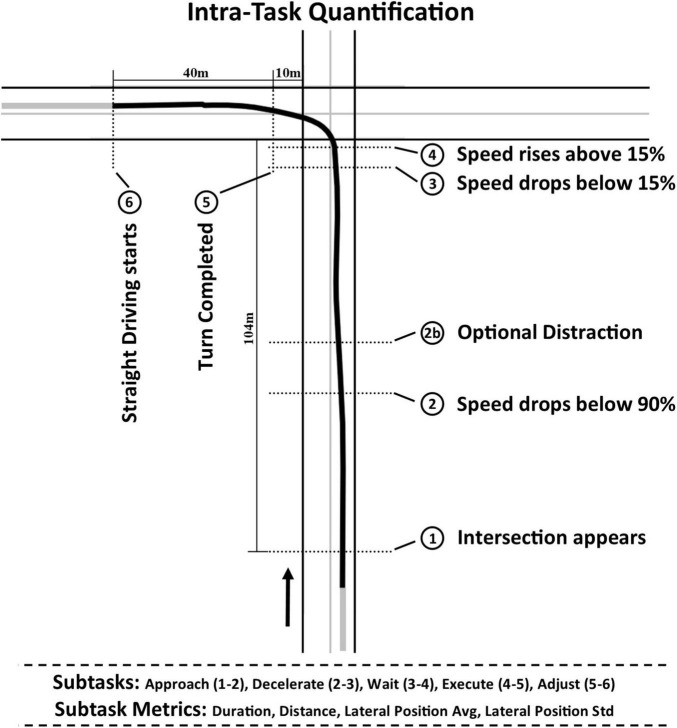
Division of intersection-based driving tasks into five subtasks: Approach (positions 1–2), Decelerate (2–3), Wait (3–4), Execute (4–5), and Adjust (5–6). For each subtask, duration, distance, lateral position average (Avg), and lateral position standard deviation (Std) metrics were computed, in addition to the stop position relative to the intersection.

Driving behavior at each intersection was also divided into five subtasks with different functional requirements (Figure 1). This division was meant to increase the opportunity, through quantification of intra-task behavior, to capture important features of driving performance that otherwise might be obscured by temporal averaging over the total task duration. The data analysis was performed using Python 3 unless explicitly stated otherwise.

The first subtask (*Approach*) began with the announcement of the task and lasted until the participant started to decelerate in preparation for the intersection, defined as a speed decrease below 90% of the average speed before the beginning of the audio cue (this value was chosen because a variability in speed of less than 10% was common during regular straight driving without indicating deceleration). During this subtask, the intersection came into sight and the participants had to evaluate the situation and plan for how they would navigate the intersection. The second subtask (*Decelerate*) was the deceleration process, defined as the interval from beginning of deceleration to when participants “came to a stop” at the intersection. For the set of distraction tasks, participants were prompted to respond to an auditory distraction during either the Approach or Decelerate subtask, depending on how early they chose to start decelerating. The third subtask (*Wait*) spanned the waiting period, either for traffic lights to change color prior to turning, for a pedestrian to cross the road, or for traffic to separate sufficiently to execute a successful left turn maneuver. During this subtask, a substantial number of participants did not come to a full stop during intersections (22% of all trials). In favor of inclusivity, a drop of speed below 15% of the average initial speed and subsequent rise above 15% were chosen to define the start and end of the Wait subtask, respectively (lower thresholds would have led to a decrease in sample size as select participants would have had too many omitted driving tasks due to excessive rolling stops). Increases in the distance traversed during the Wait subtask captured this behavior in the subsequent analysis. The stop position was defined as the time-averaged mean distance to the intersection during this subtask. The fourth subtask (*Execute*) required individuals to turn through the intersection and included their driving behavior to a distance of 10 m past the intersection (programmatically defined as the spatial end of the intersection). The fifth subtask (*Adjust*) spanned a fixed distance of 40 m after turn completion and described the transition of driving behavior back into regular straight driving which could, for example, include correcting for lane deviations and overly sharp turns. This distance was chosen to make the ends of intersection tasks coincide with the starts of straight driving tasks; visual inspection revealed that trajectory variability caused by adjusting from the turn did not extend beyond this fixed distance.

For each of the five subtasks described above, four metrics were chosen to give a detailed summary of driving behavior. For consistency and comparison with other driving literature such as (Lu and Pernía, 2000), (1) duration of the subtask and (2) total distance traveled during the subtask were recorded. Additionally, turn trajectories were compared to a reference trajectory for each individual task type which for straight road segments simply followed the middle of the lane and for turns followed the average trajectory for middle-aged drivers. The (3) time-averaged lateral displacement from the reference trajectory corresponded to whether turns and other subtasks were sharper (negative displacement) or wider (positive displacement) than the reference trajectory. Additionally, (4) standard deviation of lateral lane displacement (as a measure of root-mean-squared deviation from the mean) corresponded to whether this displacement was consistent within the subtask or subject to variability. For each intersection-based task (i.e., L, LT, LTA, R, RP), this resulted in a set of twenty behavioral metrics. Additionally, stop position was computed in terms of the distance to the intersection. Thus, *twenty-one subtask metrics* were recorded for each driving task (5 subtasks × 4 metrics + stop position). These values were averaged across the trials of each simulated driving task, per participant.

Complementing these subtask metrics, *three “total task” metrics* were recorded for the overall simulated driving behavior, including duration, time-averaged lateral lane position, and lateral lane position standard deviation, using the STISIM base software. The total distance traveled was consistent across each type of subtask and thus not considered for analysis. As straight driving tasks (i.e., S and SA) naturally did not follow the described subtask progression, only total task metrics were computed for these tasks. Total task metrics were calculated per task and per participant in the same manner as indicated above for the subtask metrics.

Analysis focussed on the age-dependence of these behavioral metrics for each driving task. The subsequent statistical analysis to characterize effects for the young, middle-aged, and old participant groups was performed independently for the set of subtask, total task, and distraction metrics for each task type (i.e., S, SA, L, LT, LTA, R, RP). Initially, Levene’s test for homogeneity of variance, as well as D’Agostino and Pearson’s test for normality were conducted. Given that these tests failed for very many of the behavioral metrics due to physical constraints imposed by the driving simulation (see section “3 Results”), age groups were compared using the omnibus non-parametric Kruskal-Wallis test on each behavioral metric. To account for the high number of behavioral metrics tested, *p*-values were corrected for multiple comparisons using the false discovery rate at a threshold of *q* < 0.05 (Benjamini and Hochberg, 1995). For behavioral measures showing significant age effects, *post-hoc* application of Dunn’s test identified pairwise differences using the middle-aged group as the reference (i.e., young versus middle-aged, old versus middle-aged) at a threshold of *p* < 0.05.

Beyond group performance, behavior-age associations were also characterized within each age group using Spearman’s rank correlations, corrected for multiple comparisons in the same manner as indicated above. In particular, significant effects (and strong trends) corresponding to improving simulated driving behavior in the young adult group and declining performance in the old adult group were of interest due to the elevated accident rates at both ends of the age spectrum, as mentioned in the Introduction (Tefft et al., 2013). The Spearman’s rank correlation results will be referred to as “age-dependent Spearman correlations” in the subsequent narrative.

## 3 Results

### 3.1 Quality assurance

All participants successfully completed the 23 regular straight driving tasks without crashes or major traffic violations. Focusing on the remaining 37 tasks of higher complexity (i.e., SA, L, LT, LTA, R, RP), traffic violations occurred at low rates including crashes, failure to stop at the intersection, or stopping mid-turn. The traffic violations were subsequently removed from the dataset and accounted for 5.5, 7.1, and 7.4% of trials for young adults, middle-aged adults, and old adults, respectively. In addition, driving tasks with faulty data recordings (multiple dropped time-points) were excluded accounting for 4.6, 2.4, and 4.0% of trials. Other behaviors that did not result in removal from analysis included sharp turns with part of the vehicle off-road or in the wrong lane (6.0, 4.0, and 3.8% of trials), and deviations from typical intersection behavior, predominantly in the form of multiple braking or acceleration attempts (7.6, 13.9, and 35.6% of trials).

Tests for normality (*p* < 0.05 using D’Agostino and Pearson’s test) revealed that turn trajectories as well as Approach durations and distances did not follow normal distributions but were predominantly skewed in favor of sharper turns and late braking. Moreover, the majority of duration and distance metrics showed inhomogeneity of variance (*p* < 0.05 using Levene’s test), with increased variance in the group of old adults. The Kruskal-Wallis test was chosen based on these observations. The most salient metric results are shown in terms of median values and interquartile ranges (IQRs) in tables and related figures below. In particular, for brevity the figures show results only for left turn tasks, which are relatively straightforward to compare as a set. Comprehensive tables of the comparisons of behavioral metrics, and age-dependent Spearman correlations, can be found in the Supplementary material.

### 3.2 Total task metrics for straight driving

Results of the group comparison of each total task metric between young adults, middle-aged adults, and old adults for straight driving tasks are listed in Table 1. Significant differences in total task metrics between age groups are indicated in the table (gray cells), after adjustment for multiple comparisons (false discovery rate q < 0.05).

**TABLE 1:** Tabulated list of total task metrics (overall duration, lateral lane position average, and lateral lane position standard deviation) across the three age-groups for regular straight driving S and distracted straight driving SA, quoted as median values and interquartile ranges.

	Young	Middle-aged	Old	Effect size
**Driving task S**				
Overall Duration (s)	10.4 (9.9; 11.2)	10.5 (9.9; 11.0)	12.3 (11.0; 13.8)	0.28
Lateral Lane Position Avg (m)	2.6 (2.4; 2.9)	2.5 (2.4; 2.7)	2.6 (2.5; 2.9)	0.01
Lateral Lane Position Std (m)	0.3 (0.3; 0.4)	0.3 (0.3; 0.4)	0.4 (0.3; 0.5)	0.06
**Driving task SA**				
Overall Duration (s)	10.3 (9.7; 10.8)	10.2 (9.7; 10.7)	12.4 (11.2; 14.1)	0.27
Lateral Lane Position Avg (m)	2.7 (2.4; 2.8)	2.7 (2.4; 2.8)	2.7 (2.4; 3.0)	0.01
Lateral Lane Position Std (m)	0.3 (0.2; 0.4)	0.4 (0.3; 0.5)	0.4 (0.3; 0.5)	0.04

The column labeled “Effect Size” reports the epsilon-squared value of the omnibus Kruskal-Wallis test; subsequent pairwise significant differences in behavioral metrics with respect to the middle-aged group, using Dunn’s test (*p* < 0.05), are highlighted in gray.

During both regular straight driving S and distracted straight driving SA, young adults showed no significant difference from middle-aged adults in Overall Duration, Lateral Lane Position Average (Avg), and Lateral Lane Position Standard Deviation (Std). For context, across both tasks the two groups completed the two tasks in median times ranging from 10.2 to 10.5 s, whereas the minimum duration was 9.6 s when driving at the speed limit of 60 km/h. Within the group of young adults, no significant age-dependent Spearman correlations were found for any of the total task metrics for the S task, however, one significant correlation was found for the SA task for young adults (see Supplementary material): the Lateral Lane Position Std [correlation coefficient (95% confidence interval) = −0.47 (−0.73: −0.10)].

**FIGURE 2:**
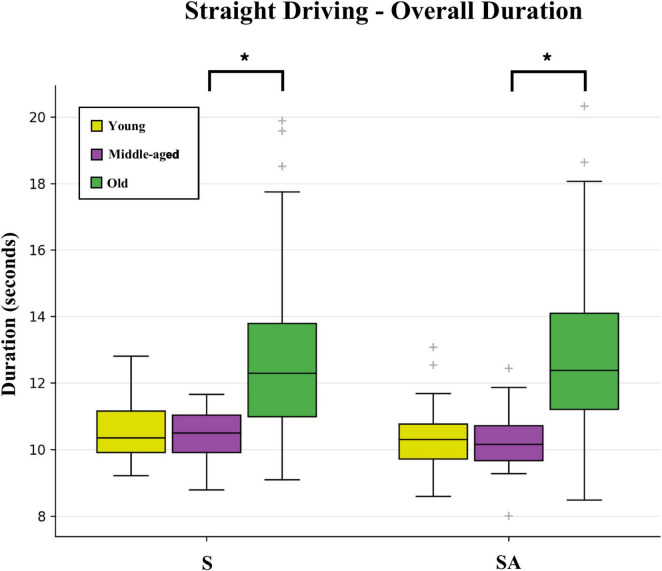
Box and whisker plot showing the distributions of Overall Duration total task metrics for the three age groups (young, middle-aged, and old adults) for regular straight driving S and distracted straight driving SA. The boxes extend from the lower to upper quartile values of the data, with a line at the median. Whiskers further include data within 1.5 times the interquartile range, with the remaining outlier data plotted as crosshairs. False discovery rate corrected significant differences (q < 0.05) are indicated by bold lines and an asterisk.

Old adults took significantly longer (Figure 2) to complete both regular straight driving S and distracted straight driving SA in comparison to middle-aged adults (median Overall Duration for S: 12.3 s versus 10.5 s; SA: 12.4 s versus 10.2 s, respectively). These values for old adults correspond to driving approximately 8 km/h below the speed limit. Furthermore, two total task metrics also showed positive age-dependent Spearman correlations for old adults (see Supplementary material): the Lateral Lane Position Avg [S: 0.44 (0.22: 0.62); SA: 0.35 (0.12: 0.55)] and Lateral Lane Position Std [S: 0.43 (0.20: 0.61); SA: 0.46 (0.24:0.63)].

### 3.3 Total task metrics for intersections

**FIGURE 3:**
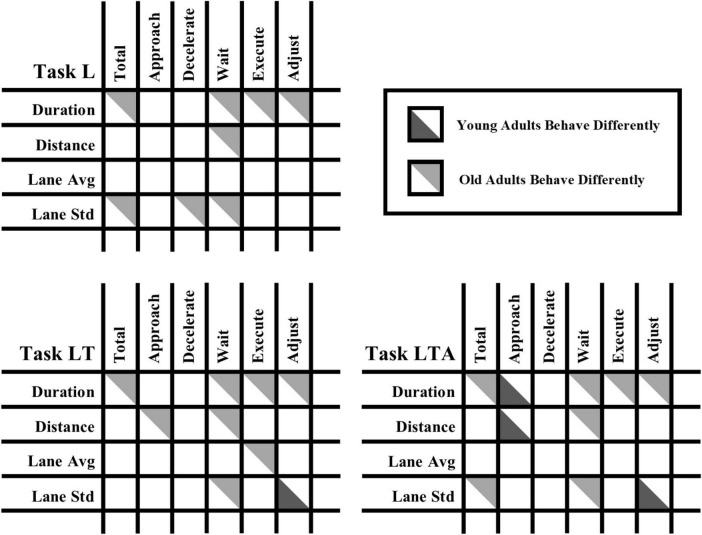
Overall tabular visualization of the significant behavioral differences for the three types of left turn tasks (basic left turns L, left turns with oncoming traffic LT, and left turns with oncoming traffic and distraction LTA) as observed by total task analysis (first column) and subtask analysis (next five columns: Approach, Decelerate, Wait, Execute, Adjust) for the four different performance metric categories investigated in the study, as listed in the rows (Duration, Distance, Lane Avg, and Lane Std). The three tables provide a complete overview of all statistically significant differences in the behavioral metrics between young adults and middle-aged adults (coded in dark gray) as well as differences between old adults and middle-aged adults (coded in light gray). The visualized differences correspond to false discovery rate-corrected significant effects between young and old adults and the middle-aged group (q < 0.05, Kruskal-Wallis test and *post-hoc* application of Dunn’s test). Lane Avg, Lateral Lane Position Average; Lane Std, Lateral Lane Position Standard Deviation. Note that overview results for the stop position are incompatible with this format and are not shown.

Results for each total task metric for the young, middle-aged, and old adults groups are listed in Table 2 for the three types of left turn tasks. Furthermore as an initial overview, Figure 3 summarizes the total task, as well as the subtask metrics described in the subsequent sections, across left turns tasks, highlighting significant behavioral differences in comparison to middle-aged drivers. Young adults showed no significant behavioral differences in total task metrics in comparison to middle-aged adults. Note that for right turn tasks, comparisons for the R task also showed no significant effects but for the RP task, young adults drove further to the right than middle-aged adults (median Lateral Lane Position Avg: 4.9 m versus 4.5 m; see Supplementary material).

**TABLE 2:** Tabulated list of total task metrics (overall duration, lateral lane position average, and lateral lane position standard deviation) for the three types of left turn tasks (basic left turns L, left turns with oncoming traffic LT, and left turns with oncoming traffic and distraction LTA) across the three age-groups, quoted as median values and interquartile ranges.

	Young	Middle-aged	Old	Effect size
**Driving task L**				
Overall Duration (s)	18.4 (17.6; 20.0)	17.6 (17.1; 20.2)	23.7 (21.0; 26.5)	0.36
Lateral Lane Position Avg (m)	−0.6 (−0.8; −0.2)	−0.3 (−0.6; −0.2)	−0.6 (−0.9; −0.3)	0.02
Lateral Lane Position Std (m)	6.9 (6.8; 7.0)	6.8 (6.7; 7.0)	7.0 (6.8; 7.4)	0.07
**Driving task LT**				
Overall Duration (s)	19.3 (18.3; 19.8)	19.2 (17.2; 22.1)	27.0 (22.8; 35.3)	0.30
Lateral Lane Position Avg (m)	−0.1 (−0.5; 0.1)	−0.1 (−0.3; 0.4)	0.3 (−0.2; 0.9)	0.07
Lateral Lane Position Std (m)	6.6 (6.4; 6.8)	6.5 (6.3; 6.8)	6.2 (5.7; 6.6)	0.07
**Driving task LTA**				
Overall Duration (s)	19.5 (18.0; 23.0)	20.1 (18.7; 23.9)	28.2 (23.6; 36.9)	0.30
Lateral Lane Position Avg (m)	−0.0 (−0.4; 0.2)	0.0 (−0.1; 0.4)	0.4 (−0.1; 0.7)	0.12
Lateral Lane Position Std (m)	6.5 (6.2; 6.6)	6.4 (6.0; 6.6)	6.0 (5.7; 6.4)	0.11

The column labeled “Effect Size” reports the epsilon-squared value of the omnibus Kruskal-Wallis test; subsequent pairwise significant differences in behavioral metrics with respect to the middle-aged group, using Dunn’s test (*p* < 0.05), are highlighted in gray. Avg, average; Std, standard deviation.

Old adults had increased median Overall Duration values when compared to middle-aged adults for each type of driving task as shown in Table 2 (L: 23.7 s versus 17.6 s; LT: 27.0 s versus 19.2 s; LTA: 28.2 s versus 20.1 s, respectively) and Supplementary material (R: 23.9 s versus 17.9 s; RP: 24.4 s versus 18.5 s, respectively). In similar comparison, old adults showed increased medial Lateral Lane Position Std values for the L task (7.0 m versus 6.8 m, respectively) and decreased values for the LTA task (6.0 m versus 6.4 m, respectively). Furthermore, old adults showed significant age-dependent Spearman correlations for Lateral Lane Position Avg for the LTA task [0.30 (0.06: 0.51)], indicating a progressive tendency to drive further to the right in the lane within this cohort.

### 3.4 Approach and Decelerate subtask metrics

The findings for the subtask metrics are reported in this and subsequent sections. For brevity, the key salient effects are shown with figures, with all tabulated values given in the Supplementary material. Summary Figure 3 also shows the predominant feature that the total task metrics report few significant effects; whereas many more subtask metrics report significant effects in the young adults, and especially in the old adults, when compared to middle-aged adults.

**FIGURE 4:**
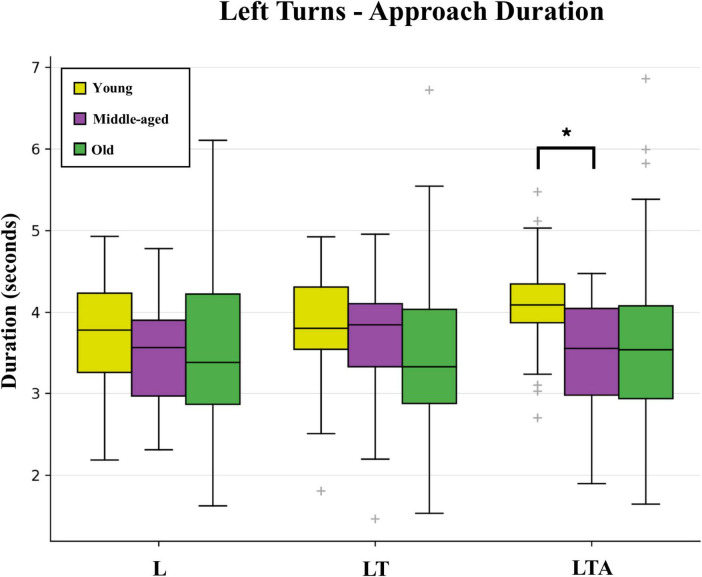
Box and whisker plot showing Approach Duration values for the three types of left turn tasks (basic left turns L, left turns with oncoming traffic LT, and left turns with oncoming traffic and distraction LTA) across the three age-groups. False discovery rate corrected significant differences (q < 0.05) are indicated by bold lines and an asterisk.

The first subtask metrics are Approach and Decelerate, for which young adults showed no significant differences compared to middle-aged adults for L, LT, R, and RP tasks. The one exception was the LTA task (see Figure 4), for which young adults took significantly longer than middle-aged adults to start decelerating when approaching the intersection (median Approach Duration: 4.1 s versus 3.6 s, respectively). This effect was similarly observed as an increase in the distance from the starting point that young adults started to decelerate during the LTA task (median Approach Distance: 63.2 m versus 55.5 m, respectively). Young adults also showed significant age-dependent Spearman correlations for Approach Lateral Lane Position Avg values [0.58 (0.24: 0.79)].

For old adults compared to middle-aged adults, however, a range of effects were found mostly for tasks that did not involve distraction. As indicated by the median Approach Distance, older adults started decelerating at a significantly earlier position for the LT task (46.6 m versus 56.4 m, respectively); the R task (47.1 m versus 51.7 m) and the RP task (46.6 m versus 54.4 m). Old adults also decelerated during right turn tasks more slowly than middle-aged adults (median Decelerate Duration for R: 4.7 s versus 3.8 s, respectively; and for RP: 4.8 s versus 3.3 s); and showed several significant age-dependent Spearman correlations, for Approach Lateral Lane Position Avg [L: 0.44 (0.21: 0.62); LT: 0.34 (0.11: 0.54); LTA: 0.36 (0.12: 0.55); R: −0.31 (−0.52: −0.07); RP: −0.62 (−0.75: −0.45)] as well as Decelerate Lateral Lane Position Avg [L: 0.40 (0.17: 0.58); LT: 0.39 (0.16: 0.58); LTA: 0.38 (0.15: 0.58); R: −0.39 (−0.58: −0.16); RP: −0.37 (−0.57: −0.14)]. These correlations indicate a tendency to drive with an increasingly rightward lane position as old age increases.

### 3.5 Stop position and Wait subtask metrics

**FIGURE 5:**
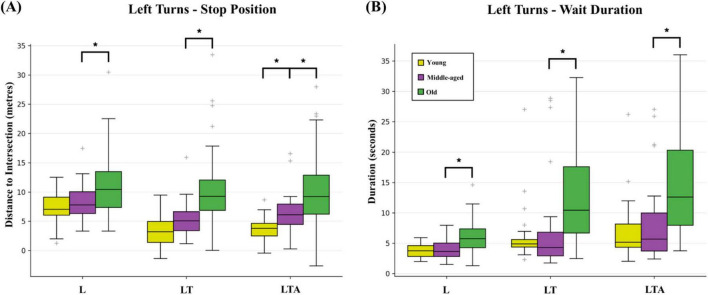
Box and whisker plot showing results for **(A)** stop position in terms of distance from the intersection, and **(B)** wait duration for the three types of left turn tasks (basic left turns L, left turns with oncoming traffic LT, and left turns with oncoming traffic and distraction LTA) across the three age-groups. False discovery rate corrected significant differences (q < 0.05) are indicated by bold lines and an asterisk.

Young adults mostly did not show significant differences when compared to middle-aged adults in terms of Stop Position and Wait subtask metrics. However, young adults did stop significantly closer to the intersection as captured by the median Stop Position on two tasks: LTA (3.8 m versus 6.1 m, respectively) as shown in Figure 5A, and RP (6.1 m versus 8.0 m, respectively). As well, young adults had a significantly lower Wait Lateral Lane Position Avg for the RP task (−0.3 m versus 0.2 m, respectively).

Conversely, old adults showed many more pronounced Stop Position and Wait effects compared to middle-aged adults. As shown in Figure 5A, the median Stop Position was increased (i.e., further from the intersection) for old adults for each of the left turn tasks (L: 10.4 versus 7.8 m, respectively; LT: 9.3 m versus 5.1 m; LTA: 9.2 m versus 6.1 m) but not for right turn tasks. No significant age-dependent Spearman correlations for median Stop Position remained after false discovery rate correction. Across all turning tasks, old adults showed significantly increased median Wait Durations at the intersection compared to middle-aged adults, as shown in Figure 5B (L: 5.8 s versus 3.7 s, respectively; LT: 10.5 s versus 4.3 s; LTA: 12.6 s versus 5.7 s). For right turns, the analogous values were R: 6.0 s versus 3.9 s; RP: 6.3 s versus 4.2 s. Compared to middle-aged adults, old adults also showed increased median Wait Distance values across all tasks (L: 7.8 m versus 3.4 m; LT: 10.3 m versus 4.6 m; LTA: 10.8 m versus 3.4 m; R: 8.1 m versus 3.4 m; RP: 7.8 m versus 5.2 m), indicative of less abrupt braking and rolling stops. Furthermore, old adults also showed elevated median values of Wait Lane Position Std, for all tasks except RP; and for LT, LTA, and R there were significant age-dependent Spearman correlations for Wait Distance, Wait Lane Position Avg, and Wait Lane Position Std (see Supplementary material).

### 3.6 Execute and Adjust subtask metrics

**FIGURE 6:**
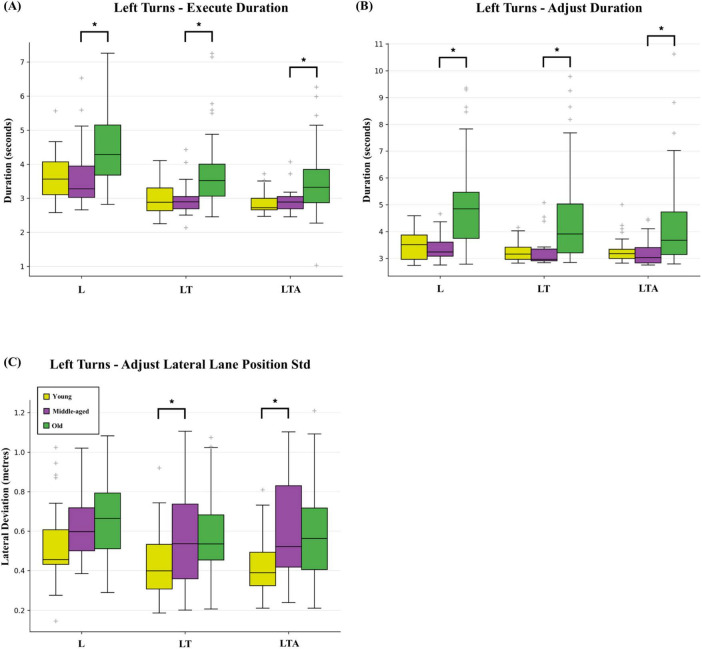
Box and whisker plot showing results for **(A)** Execute Duration, **(B)** Adjust Duration, and **(C)** Adjust Lateral Lane Position standard deviation (Std) for the three types of left turn tasks (basic left turns L, left turns with oncoming traffic LT, and left turns with oncoming traffic and distraction LTA) across the three age-groups. False discovery rate corrected significant differences (q < 0.05) are indicated by bold lines and an asterisk.

Turning now to the turn execution and transition back to straight driving, young adults showed very few significant differences in Execute and Adjust subtask metrics when compared to middle-aged adults. Decreased median values for Adjust Lateral Lane Position Std were observed for two left turn tasks, LT (0.40 m versus 0.54 m, respectively) and LTA (0.39 m versus 0.52 m, respectively) as shown in Figure 6C. Young adults also showed significant age-dependent Spearman correlations for Execute Lateral Lane Position Avg values [0.57 (0.23:0.79)], indicating the tendency to drive wider turns as age increases.

Again, old adults showed many more significant effects across multiple subtask metrics in comparison to middle-aged adults. Median Execute Duration values were significantly elevated across all tasks (left turn tasks shown in Figure 6A, L: 4.3 s versus 3.3 s, respectively; LT: 3.5 s versus 2.9 s; LTA: 3.3 s versus 2.9 s; R: 3.0 s vs. 2.8 s; RP: 3.2 s vs. 2.6 s). Similar effects were also found for the median Adjust Duration (left turn tasks shown in Figure 6B, L: 4.9 s versus 3.2 s; LT: 3.9 s versus 3.0 s; LTA: 3.7 s versus 3.0 s; R: 4.8 s versus 3.7 s; RP: 4.8 s versus 3.6 s). Moreover, old adults consistently showed significant age-dependent Spearman correlations, consistent with increasing turn radius when performing left turns [Execute Lateral Lane Position Avg for L: 0.47 (0.26: 0.64); LT: 0.50 (0.29: 0.66); LTA: 0.49 (0.28: 0.66)] and decreasing turn radius when performing the RP task [−0.43 (−0.61: −0.21)]. A similar age-dependent Spearman correlation was also observed for Adjust Lateral Lane Position Avg values in the case of left turns [L: 0.34 (0.11: 0.54); LT: 0.56 (0.37: 0.71); LTA: 0.55 (0.35: 0.70)].

## 4 Discussion

The present study provides new findings regarding simulated driving behavior, based on a large dataset consisting of young, middle-aged, and old adults, who were assessed on various tasks including straight driving, making turns, and driving with distraction. In addition to replicating and extending the results of previous simulated driving studies, the results support the two hypotheses of interest. First, by dividing simulated driving tasks into shorter-duration subcomponents (subtasks), and by developing and applying subtask metrics, nuanced differences in simulated driving behavior were found between young adults compared to middle-aged adults, and between old adults and middle-aged adults. The subtask metrics provided enhanced detection sensitivity compared to those used more traditionally to quantify behavior over the whole duration of a given driving task. Second, young adults and old adults each showed a different set of subtasks, across the range of tasks administered, for which there were significant within-group age-dependent Spearman correlations observed for driving behavior. The details and implications of these findings are discussed below, along with the scientific limitations of the work and perspectives on future work to be undertaken in this area of research.

Considering first the results over all simulated driving tasks, as captured by the total task metrics, the most striking observation was that young adults did not show significant effects when compared to middle-aged adults and only showed one within-group age-dependent Spearman correlation for Lateral Lane Position Std for the straight driving with distraction SA task. Conversely, old adults showed multiple effects. For young adults, the one significant correlation effect is consistent with the expected gains in driving ability and ability to deal with distractions that come with increasing driving experience and brain maturation with age in this cohort (as discussed further below). Yet it is surprising at first glance, based on the same reasoning, that similar effects were not observed for young adults performing any of the turning tasks. For old adults in comparison to middle-aged adults, the Overall Duration was increased irrespective of the task, a finding that is consistent with prior studies (Shinar et al., 2005; Becic et al., 2007; Doroudgar et al., 2017; Michaels et al., 2017; Robertsen et al., 2022). For the straight driving S and SA tasks, it could be straightforwardly inferred from the results that the longer duration was due to old adults driving approximately 8 km/h below the 60 km/h speed limit. However, interpretation of the total task duration and average speed effects is more challenging for all the other tasks (L, LT, LTA, R, RP) as they are considerably more complex, involving acceleration, deceleration, waiting, and turning. Qualitatively, these latter tasks were completed in longer durations than the S and SA tasks, with the most challenging tasks (LT, LTA) showing the longest durations. As no instructions on speed were given as part of the experiment other than to follow normal traffic laws, possibly the behavior of old adults reflected a conscious choice to engage in more careful driving strategies even for basic tasks, as suggested in prior literature (Michaels et al., 2017; Pope et al., 2017; Robertsen et al., 2022) - likely with a level of caution that modulated with the perceived challenge or risk. This is plausible but difficult to interpret further without more behavioral performance details. Additional effects were also observed inconsistently across tasks for old adults both in comparison with middle-aged adults and as within-group correlations (increases and decreases in median Lateral Lane Position Std for the L task and LTA task, respectively; and age-dependent Spearman correlations for Lateral Lane Position Std for S and SA tasks, and between age and Lateral Lane Position Avg for the LTA task). Evidently the simulated driving behavior of old adults is characterized by more than simply slower overall performance, pointing to the need for subtask investigations and analyses where possible, and to search for commonalities and differences between the various subcomponents of different driving tasks.

As expected, further significant effects were identified when subtask analyses were conducted for each of the young adult and old adult groups in comparison to middle-aged adults. For young adults, there were differences in the Lateral Lane Position Avg during the Wait subtask (further to the left for the RP task), the Lateral Lane Position Std during the Adjust subtask (lower for the LT and LTA tasks), and in the Stop Position at the intersection (closer to the intersection for the LTA and RP tasks), as well as the Duration and Distance during the Approach subtask (both significantly higher for the LTA task). Many of these effects are suggestive of less driving experience and skill in the young adults group, regarding positioning the car at the intersection in more challenging turn situations (LTA and RP) and less variability in returning to straight driving after crossing through traffic (LT and LTA), whereas middle-aged adults dynamically changed their trajectory to match the situation at hand. In particular, young adults came to a halt significantly closer to the intersection for LTA and RP tasks involving traffic and pedestrians – which has previously been suggested as an indicator for at-risk driving (Michaels et al., 2017).

The subtask metrics also provided increased insight into the significant changes in driving behavior executed by young adults when they were distracted during the LTA task. Compared to middle-aged adults, young adults showed significantly delayed reactions to the appearance of an intersection, as quantified by Approach subtask Duration and Approach Distance values. A reasonable interpretation for the observed effects is that while young adults were attempting to answer the questions during driving, the behavioral difference reflected a reallocation of mental resources – which is consistent with the known safety implications for this group (Strayer and Drew, 2004; Zhang et al., 2019). “Internal distractions,” such as responding to passengers in the car or operating smartphones form a substantial contribution to the elevated accident rate of young drivers, as confirmed by reports of the American Automobile Association (Carney et al., 2015). Regarding the alleviation of risky driving behavior in young adults as they gain years and driving experience, it was also notable that the value of one metric for the SA task (Lateral Lane Position Std) and values of two subtask metrics for the LTA task (Approach Lateral Lane Position Avg and Execute Lateral Lane Position Avg) showed strong correlation with age for young adults – consistent with how simulated driving behavior with distraction became increasingly similar to that of middle-aged adults. Overall, the differences in driving behavior displayed by young adults in comparison to middle-aged adults are consistent with literature on brain development and complex skill acquisition in the final stages of maturation to adulthood (Arain et al., 2013).

Turning now to the simulated driving behavior of old adults in comparison to that of middle-aged adults, many effects were revealed by the subtask analysis. Similar to the reduced speed with which old adults performed the S and SA tasks, there was a reduction in speed during the Approach subtask for three out of five turning conditions (LT, R, RP) – with each showing no difference between groups in the Approach Duration values, but a significant reduction in the Approach Distance values indicating that they came to a halt earlier and more gradually (note that speed = distance/duration). There were also speed reductions across all turning tasks during the Execution and Adjust subtasks, but this time the effect was reversed: the turning and adjusting to normal straight driving took longer to complete by old adults whereas the distance associated with each subtask was not significantly different from middle-aged adults, noting that the Adjust subtask is fixed in distance by design. In addition, older adults had characteristically different behavior during the Wait subtask prior to turn execution, characterized by elevated Wait Duration, Wait Distance, and Wait Lateral Lane Position Std (i.e., primarily rolling stop behavior). This was observed for all turning tasks (except for the RP task, where the lateral lane position variability effect was absent) and is characterized better as “lingering” prior to executing turns – sometimes for many more seconds than middle-aged adults, particularly during the LT and especially the LTA tasks. Furthermore, the Stop Position subtask metric showed effects for several turning tasks (L, LT, and LTA) indicating that old adults stopped further from the intersection than middle-aged adults. Last, there were characteristic within-group features of driving behavior for old adults that were observed as significant age-dependent Spearman correlations of subtask metric values. The predominant feature of this type involved Lateral Lane Position Avg, which showed significant correlations with age during Approach and Decelerate (all turning tasks), Execute (L, LTA, RP) and Adjust (L, LT, and LTA). An additional feature of this type was observed for the Wait subtask only, with significant correlations occurring for Distance, Lateral Lane Position Avg, and Lateral Lane Position Std (L, LT, and R).

Summarizing and synthesizing these findings, it is evident from the detailed observations in the present study that even when performing relatively innocuous driving maneuvers without overt error, old adults show behaviors that tend to set them apart from middle-aged adults. Old adults often drive more slowly, start decelerating toward intersections earlier, and stop further from intersections – where they wait longer to decide and act, especially when distracted. These behaviors confirm and support the observations of multiple other groups involved in simulated driving studies as well as those based on field reports of actual driving (Dale et al., 1999; Lu and Pernía, 2000; Strayer and Drew, 2004; Shinar et al., 2005; Becic et al., 2007; Andrews and Westerman, 2012; Pope et al., 2017; Getzmann et al., 2018; Arafa et al., 2020). The consistent interpretation from this literature, and also from the present study, is that old adults are likely adopting cautious driving strategies in an attempt to compensate for age-related declines in mental processing (Brouwer and Ponds, 1994; Anstey et al., 2005), which was anecdotally confirmed by several drivers in the post-experiment questionnaire. This argument is reasonable, given other work that has shown age-related declines in healthy elderly adults in multiple domains including sensory, motor, and cognitive processing (Manard et al., 2014; Humes, 2015; Wu et al., 2021). Furthermore, the present study shows that another key aspect of the altered driving behavior in the elderly is their increasing tendency with age to stray from a centered position in their driving lane, and to make turning maneuvers with different turn radii than those of middle-aged adults. Whereas difficulties in lane maintenance with old age have been extensively described in prior literature (Shechtman et al., 2007; Bunce et al., 2012; Sun et al., 2018), no comparable systematic changes in turn trajectory have been reported to the best of our knowledge. This behavior may also be a strategy of cautiousness under the perception that it partially mitigates risks to the driver (e.g., from oncoming traffic). However, other underlying mechanisms are likely also possible, such as age-related decline in spatial attention and processing (Laurin et al., 2024).

There are a number of limitations to the present study that should be considered when interpreting the results. First, there is a concern about ecological validity, as the study does not report the behavior of actual driving, opting instead for data recording from a driving simulator that was specially constructed to permit simultaneous study of simulated driving behavior and the underlying brain activity as measured by fMRI. Importantly and as mentioned above, the results obtained with this simulator are broadly consistent with those from other simulated driving studies and field observations of actual driving behavior, suggesting that the ecological validity of the simulator is very good for the participants investigated. Nevertheless, minor deficiencies between real-world and the present simulated driving performance certainly still remain. Considering haptic feedback (representation of touch and proprioception), for example, the simulator contains a steering wheel and foot pedals (Kan et al., 2012), a substantial improvement from previous fMRI-compatible simulators that were operated by joysticks (Spiers and Maguire, 2007). Although the steering wheel and pedals are more like those from video games than those in automotive vehicles, this is likely of minor concern given that participants were given ample time to train to proficiency, transferring their driving skills from the real world to the simulator environment. Of more concern is that participants operated the simulator while they lay prone in the magnet bore (during fMRI, with analysis of the brain/behavior relationships to be reported in a future study) and lacked proprioceptive, inertial feedback during acceleration and deceleration. “Simulated driving while lying” is necessary during such imaging but is an obvious departure from naturalistic driving behavior. Due to this lack of feedback, performance may have worsened in comparison to driving in the real world, and this would be useful to investigate in future research. Interestingly, the prone position may help to mitigate the sensory conflict and cybersickness that can occur when some individuals use driving simulators while sitting upright (Gahlinger, 2006). The rate of cybersickness was very low in the present study (only a single participant reported sickness due to the simulation) and thus is judged not to have biased the results. Considering other simulator-related factors, the confines of the magnet bore did not allow for the peripheral vision component of driving tasks to be simulated and evaluated. Positions of the foot pedal and steering wheels also had to be adjusted from where they would normally be found in a car, again due to spatial restrictions, and participants were instructed to perform simulated driving maneuvers while keeping head movements to a minimum to suppress motion-related errors in the fMRI data. To mitigate these concerns, all participants had a lengthy training period (1 h) for skill transfer and acclimatization to driving with the simulator. The training duration was chosen such that learning effects could be neglected for all participants at the time of actual behavioral testing – not just for young drivers familiar with virtual reality and driving video games (Wayne and Miller, 2018). Participants reported gaining sufficient familiarity with the driving simulator after the training period, however, multiple old adults had difficulties judging distances and thus drove more cautiously to avoid potential traffic violations. Moreover, a small number of old adults intentionally only used rolling stops as they were hesitant about using the brake pedal correctly.

Related to the last point of discussion, studies have shown how different types of distractions affect certain age groups to different degrees (Stojan and Voelcker-Rehage, 2021). Thus, there could be concerns over group-related performance bias related to how the SA and LTA tasks were designed. Previous studies have shown that older adults struggled with distractions involving interacting with electronic devices within the car, whereas young adults were prone to becoming distracted by trying to formulate arguments, similar to how teen drivers are statistically worse at driving when talking to other passengers in the car (Tefft et al., 2013; Carney et al., 2015; Ouimet et al., 2015; Zhang et al., 2019). In addition to the simulator training provided to all participants, the distraction chosen for this study required both critical thinking as well as interacting with an electronic device, in an attempt to model realistic behavior affecting both ends of the age spectrum. Other potential sources that could have differentially affected the behavior of the young, middle-aged, and old adult groups include sampling bias (how representative each group was of the population at large) and, due to the limited availability of the MRI system for conducting simulated driving studies, age-dependent circadian rhythm effects on cognition (Schmidt et al., 2012; Zhang et al., 2014). Due to participant compliance and financial cost, use of the MRI system was also limited to 1 h per participant (including setup and removal of the driving simulator from the magnet) which restricted the number of driving tasks that each participant could complete. Acquisition of driving performance data over a longer duration per participant could possibly reveal additional statistically significant effects at the group level, beyond those reported here – and possibly could permit detection of effects at the participant level, which was not attempted in the present work due to statistical power considerations (and brevity). Future work could enable more lengthy data collection by conducting imaging sessions per participant on multiple days, for example – as might be useful to investigate patient participants with specific neural deficits.

The hypotheses and analyses of simulated driving results in the present work predominantly focus on differences in behavior of young adults and old adults in comparison to middle-aged adults. Middle-aged adults are the most logical initial comparator group, as they have extensive experience driving, exhibit much safer driving on average than both young and old adults, and likely have more efficient, stable activation of neural networks related to driving that are neither affected by the processes of brain maturation in young adults, nor the changes to brain function that accompany old age. Beyond comparisons with the middle-aged group, other comparisons would be interesting to report from this large dataset, including the differences between young and old adults. For brevity, such investigations are left to future studies. As this decision has been made in part due to the fact that conducting subtask analyses on multiple different driving conditions yields a large number of results, future work should also focus on efficiently quantifying the most essential features in the data. It is very likely that there are interdependencies between subtask metric values and as a consequence, it would be useful to develop multivariate data reduction strategies - for example, to determine which combinations of subtask metrics over different driving conditions elucidate the characteristic features of the simulated driving behaviors of the three age groups. Such work would be an important supplement to the descriptive, more qualitative approach taken here.

Ultimately, the ideas just mentioned will likely be best undertaken in the context of a series of fMRI studies reporting on the brain activity associated with simulated driving. Such work is in progress and promises to provide important new information, including the “core” network across multiple driving tasks executed by adults across the age-span; how nodes in this network are modulated by development, experience, and aging; how the regional brain activity in individuals links to aspects of their driving performance; how typical cognitive assessment tools engage brain areas with functional roles in simulated driving; and the extent to which simultaneous simulated driving and fMRI can be used to discriminate individuals with risky driving behavior from those with safer behavior – as well as to discriminate healthy individuals from those with various neurological or psychiatric conditions.

In conclusion, the presented results show how young adults behaved similarly to middle-aged adults during simulated driving, but with some differences especially in fine spatial control and increased susceptibility to auditory distraction. Old adults, on the other hand, showed much more different driving behavior compared to middle-aged adults by driving more cautiously and prioritizing safe driving over responding to distractions as fast as possible, while also requiring more time to complete less complex driving maneuvers. This work, involving a large study cohort and careful characterization of intra-task behavior, serves as an important benchmark of simulated driving performance measures – to inform neuroscientists and clinicians concerned with future development of better behavioral assessment tools to assess fitness-to-drive, as well as those who are interested in developing measures and assistive technologies to make driving safer. The work also lays the groundwork for future fMRI studies of the brain and behavior relationships observed from the study participants.

## Data Availability

The raw data supporting the conclusions of this article will be made available by the authors, without undue reservation.
