# Supplementary material for: Simulated driving behavior over the adult age span
Source: Front Aging Neurosci. 2025 Feb 19;17:1496224. doi: 10.3389/fnagi.2025.1496224 (PMC11879975; doi:10.3389/fnagi.2025.1496224)
Supplement: Supplementary file 1 [file Table_1.docx]

# Supplementary Materials

|  | **Young** | **Middle-Aged** | **Old** |
| --- | --- | --- | --- |
|  | **Driving Task L** | | |
| *Approach Duration (s)* | 3.8 (3.3; 4.2) | 3.6 (3.0; 3.9) | 3.4 (2.9; 4.2) |
| *Approach Distance (m)* | 55.9 (49.1; 62.5) | 52.9 (47.4; 59.6) | 48.0 (37.1; 54.6) |
| *Approach Lateral Lane Position Avg (m)* | -0.0 (-0.3; 0.3) | 0.0 (-0.2; 0.3) | 0.1 (-0.2; 0.4) |
| *Approach Lateral Lane Position Std (m)* | 0.1 (0.1; 0.2) | 0.1 (0.1; 0.2) | 0.1 (0.1; 0.2) |
| *Decelerate Duration (s)* | 3.5 (2.6; 4.2) | 3.8 (3.0; 4.1) | 4.1 (3.2; 5.9) |
| *Decelerate Distance (m)* | 29.2 (22.1; 33.4) | 30.1 (23.7; 34.9) | 30.1 (24.1; 36.5) |
| *Decelerate Lateral Lane Position Avg (m)* | -0.1 (-0.4; 0.1) | -0.1 (-0.3; 0.2) | -0.1 (-0.3; 0.2) |
| *Decelerate Lateral Lane Position Std (m)* | 0.1 (0.1; 0.2) | 0.1 (0.1; 0.1) | 0.1 (0.1; 0.2) |
| *Wait Duration (s)* | 3.8 (2.8; 4.6) | 3.7 (2.8; 5.1) | 5.8 (4.3; 7.4) |
| *Wait Distance (m)* | 2.6 (1.8; 3.5) | 3.4 (2.1; 5.0) | 7.8 (3.9; 12.7) |
| *Wait Lateral Lane Position Avg (m)* | -0.2 (-0.5; 0.1) | -0.2 (-0.4; 0.0) | -0.3 (-0.5; -0.1) |
| *Wait Lateral Lane Position Std (m)* | 0.0 (0.0; 0.0) | 0.0 (0.0; 0.1) | 0.1 (0.0; 0.2) |
| *Execute Duration (s)* | 3.6 (3.1; 4.1) | 3.3 (3.0; 3.9) | 4.3 (3.7; 5.2) |
| *Execute Distance (m)* | 19.3 (18.1; 20.2) | 18.8 (17.7; 20.3) | 18.4 (17.4; 20.5) |
| *Execute Lateral Lane Position Avg (m)* | 0.2 (-0.2; 0.4) | 0.2 (-0.2; 0.3) | 0.3 (0.1; 0.6) |
| *Execute Lateral Lane Position Std (m)* | 0.5 (0.4; 0.6) | 0.5 (0.4; 0.6) | 0.5 (0.4; 0.6) |
| *Adjust Duration (s)* | 3.5 (3.0; 3.9) | 3.2 (3.1; 3.6) | 4.9 (3.7; 5.5) |
| *Adjust Lateral Lane Position Avg (m)* | 0.2 (-0.0; 0.5) | 0.1 (-0.6; 0.4) | -0.1 (-0.3; 0.3) |
| *Adjust Lateral Lane Position Std (m)* | 0.5 (0.4; 0.6) | 0.6 (0.5; 0.7) | 0.7 (0.5; 0.8) |
| *Stop Position (m)* | 7.0 (6.0; 9.1) | 7.8 (6.4; 10.1) | 10.4 (7.4; 13.5) |
|  | **Driving Task LT** | | |
| *Approach Duration (s)* | 3.8 (3.5; 4.3) | 3.8 (3.3; 4.1) | 3.3 (2.9; 4.0) |
| *Approach Distance (m)* | 59.3 (54.3; 62.7) | 56.4 (49.2; 63.6) | 46.6 (39.6; 54.8) |
| *Approach Lateral Lane Position Avg (m)* | 0.3 (-0.1; 0.4) | 0.0 (-0.1; 0.3) | 0.1 (-0.0; 0.5) |
| *Approach Lateral Lane Position Std (m)* | 0.2 (0.1; 0.2) | 0.1 (0.1; 0.2) | 0.2 (0.1; 0.2) |
| *Decelerate Duration (s)* | 3.6 (2.8; 4.3) | 3.4 (2.7; 4.6) | 4.4 (3.1; 5.7) |
| *Decelerate Distance (m)* | 30.3 (23.7; 37.4) | 25.7 (23.4; 35.0) | 30.5 (25.3; 35.7) |
| *Decelerate Lateral Lane Position Avg (m)* | -0.1 (-0.2; 0.2) | -0.0 (-0.2; 0.1) | -0.0 (-0.3; 0.3) |
| *Decelerate Lateral Lane Position Std (m)* | 0.2 (0.1; 0.2) | 0.1 (0.1; 0.1) | 0.1 (0.1; 0.2) |
| *Wait Duration (s)* | 4.9 (4.4; 5.6) | 4.3 (2.9; 6.8) | 10.5 (6.7; 17.6) |
| *Wait Distance (m)* | 3.0 (2.5; 4.2) | 4.6 (2.7; 6.1) | 10.3 (6.9; 17.8) |
| *Wait Lateral Lane Position Avg (m)* | -0.4 (-0.6; -0.2) | -0.1 (-0.4; 0.1) | -0.1 (-0.4; 0.1) |
| *Wait Lateral Lane Position Std (m)* | 0.0 (0.0; 0.1) | 0.0 (0.0; 0.1) | 0.1 (0.0; 0.1) |
| *Execute Duration (s)* | 2.9 (2.6; 3.3) | 2.9 (2.7; 3.1) | 3.5 (3.1; 4.0) |
| *Execute Distance (m)* | 16.2 (14.8; 17.4) | 16.5 (15.4; 17.6) | 16.8 (16.0; 18.6) |
| *Execute Lateral Lane Position Avg (m)* | 0.3 (-0.2; 0.6) | 0.1 (-0.1; 0.4) | 0.4 (0.2; 0.8) |
| *Execute Lateral Lane Position Std (m)* | 0.4 (0.3; 0.5) | 0.4 (0.3; 0.6) | 0.5 (0.4; 0.6) |
| *Adjust Duration (s)* | 3.2 (3.0; 3.4) | 3.0 (2.9; 3.4) | 3.9 (3.2; 5.0) |
| *Adjust Lateral Lane Position Avg (m)* | 0.2 (-0.3; 0.4) | -0.3 (-0.8; 0.0) | -0.0 (-0.3; 0.5) |
| *Adjust Lateral Lane Position Std (m)* | 0.4 (0.3; 0.5) | 0.5 (0.4; 0.7) | 0.5 (0.5; 0.7) |
| *Stop Position (m)* | 3.2 (1.4; 5.0) | 5.1 (3.4; 6.7) | 9.3 (6.9; 12.1) |
|  | **Driving Task LTA** | | |
| *Approach Duration (s)* | 4.1 (3.9; 4.3) | 3.6 (3.0; 4.0) | 3.5 (2.9; 4.1) |
| *Approach Distance (m)* | 63.2 (58.2; 65.7) | 55.5 (47.9; 61.3) | 47.7 (41.6; 57.3) |
| *Approach Lateral Lane Position Avg (m)* | -0.0 (-0.3; 0.3) | 0.1 (-0.2; 0.3) | 0.1 (-0.2; 0.4) |
| *Approach Lateral Lane Position Std (m)* | 0.2 (0.1; 0.2) | 0.1 (0.1; 0.2) | 0.2 (0.1; 0.2) |
| *Decelerate Duration (s)* | 2.9 (2.5; 3.6) | 3.7 (2.7; 5.1) | 3.7 (2.9; 5.2) |
| *Decelerate Distance (m)* | 24.8 (21.3; 30.5) | 28.6 (24.4; 36.2) | 28.2 (21.8; 33.0) |
| *Decelerate Lateral Lane Position Avg (m)* | -0.2 (-0.5; 0.0) | -0.0 (-0.2; 0.2) | -0.1 (-0.3; 0.3) |
| *Decelerate Lateral Lane Position Std (m)* | 0.1 (0.1; 0.2) | 0.1 (0.1; 0.1) | 0.1 (0.1; 0.1) |
| *Wait Duration (s)* | 5.2 (4.3; 8.2) | 5.7 (3.7; 10.0) | 12.6 (8.0; 20.3) |
| *Wait Distance (m)* | 3.2 (2.6; 4.5) | 3.4 (2.7; 7.0) | 10.8 (7.1; 19.0) |
| *Wait Lateral Lane Position Avg (m)* | -0.5 (-0.6; -0.2) | -0.2 (-0.3; 0.1) | -0.2 (-0.5; 0.1) |
| *Wait Lateral Lane Position Std (m)* | 0.0 (0.0; 0.1) | 0.0 (0.0; 0.1) | 0.1 (0.0; 0.1) |
| *Execute Duration (s)* | 2.7 (2.7; 3.0) | 2.9 (2.7; 3.1) | 3.3 (2.9; 3.8) |
| *Execute Distance (m)* | 16.6 (15.0; 17.0) | 17.0 (15.8; 17.9) | 16.7 (15.7; 18.2) |
| *Execute Lateral Lane Position Avg (m)* | 0.5 (0.0; 0.8) | 0.4 (-0.0; 0.6) | 0.6 (0.3; 1.0) |
| *Execute Lateral Lane Position Std (m)* | 0.5 (0.4; 0.7) | 0.6 (0.4; 0.7) | 0.6 (0.5; 0.8) |
| *Adjust Duration (s)* | 3.2 (3.0; 3.3) | 3.0 (2.8; 3.4) | 3.7 (3.1; 4.7) |
| *Adjust Lateral Lane Position Avg (m)* | 0.0 (-0.3; 0.5) | -0.3 (-0.9; 0.3) | -0.1 (-0.4; 0.4) |
| *Adjust Lateral Lane Position Std (m)* | 0.4 (0.3; 0.5) | 0.5 (0.4; 0.8) | 0.6 (0.4; 0.7) |
| *Stop Position (m)* | 3.8 (2.5; 4.7) | 6.1 (4.5; 8.0) | 9.2 (6.2; 12.9) |
|  | **Driving Task R** | | |
| *Approach Duration (s)* | 3.7 (3.3; 4.0) | 3.5 (3.0; 3.9) | 3.3 (2.7; 3.8) |
| *Approach Distance (m)* | 55.3 (48.0; 61.6) | 51.7 (46.8; 58.9) | 47.1 (40.4; 51.9) |
| *Approach Lateral Lane Position Avg (m)* | -0.2 (-0.4; -0.0) | -0.1 (-0.3; -0.0) | -0.3 (-0.7; 0.1) |
| *Approach Lateral Lane Position Std (m)* | 0.1 (0.1; 0.2) | 0.1 (0.1; 0.2) | 0.2 (0.1; 0.2) |
| *Decelerate Duration (s)* | 3.3 (2.7; 4.6) | 3.8 (2.8; 4.3) | 4.7 (3.7; 5.9) |
| *Decelerate Distance (m)* | 28.6 (23.8; 36.3) | 30.1 (25.4; 35.8) | 32.8 (26.7; 38.9) |
| *Decelerate Lateral Lane Position Avg (m)* | -0.3 (-0.7; -0.0) | -0.0 (-0.3; 0.1) | -0.3 (-0.6; 0.2) |
| *Decelerate Lateral Lane Position Std (m)* | 0.1 (0.1; 0.1) | 0.1 (0.1; 0.2) | 0.2 (0.1; 0.2) |
| *Wait Duration (s)* | 3.7 (3.1; 5.0) | 3.9 (2.9; 4.9) | 6.0 (4.4; 9.1) |
| *Wait Distance (m)* | 2.6 (1.9; 3.9) | 3.4 (2.4; 6.0) | 8.1 (4.3; 12.6) |
| *Wait Lateral Lane Position Avg (m)* | -0.4 (-0.7; -0.1) | -0.1 (-0.4; 0.1) | -0.2 (-0.6; 0.1) |
| *Wait Lateral Lane Position Std (m)* | 0.0 (0.0; 0.0) | 0.0 (0.0; 0.1) | 0.1 (0.1; 0.3) |
| *Execute Duration (s)* | 2.6 (2.4; 2.8) | 2.8 (2.3; 3.0) | 3.0 (2.6; 3.5) |
| *Execute Distance (m)* | 11.4 (10.5; 13.3) | 11.8 (10.5; 13.4) | 11.6 (10.6; 12.3) |
| *Execute Lateral Lane Position Avg (m)* | -0.2 (-0.4; 0.1) | 0.1 (-0.1; 0.2) | 0.2 (-0.1; 0.4) |
| *Execute Lateral Lane Position Std (m)* | 0.4 (0.3; 0.6) | 0.4 (0.3; 0.5) | 0.4 (0.3; 0.6) |
| *Adjust Duration (s)* | 3.6 (3.3; 4.2) | 3.7 (3.6; 4.3) | 4.8 (4.1; 5.9) |
| *Adjust Lateral Lane Position Avg (m)* | 0.3 (0.1; 0.7) | 0.5 (0.1; 0.8) | 0.7 (0.3; 1.2) |
| *Adjust Lateral Lane Position Std (m)* | 0.5 (0.4; 0.7) | 0.5 (0.4; 0.6) | 0.5 (0.3; 0.6) |
| *Stop Position (m)* | 6.8 (6.1; 8.1) | 7.8 (6.0; 9.3) | 9.5 (6.8; 12.5) |
|  | **Driving Task RP** | | |
| *Approach Duration (s)* | 4.0 (3.3; 4.2) | 3.6 (3.2; 4.0) | 3.5 (2.9; 4.1) |
| *Approach Distance (m)* | 59.0 (49.5; 67.3) | 54.4 (51.8; 60.4) | 46.6 (39.4; 57.1) |
| *Approach Lateral Lane Position Avg (m)* | -0.0 (-0.3; 0.3) | 0.1 (-0.4; 0.3) | -0.2 (-0.4; 0.1) |
| *Approach Lateral Lane Position Std (m)* | 0.2 (0.1; 0.2) | 0.1 (0.1; 0.2) | 0.2 (0.1; 0.2) |
| *Decelerate Duration (s)* | 3.5 (2.3; 3.8) | 3.3 (2.7; 4.6) | 4.8 (3.1; 5.9) |
| *Decelerate Distance (m)* | 26.6 (20.2; 33.2) | 26.6 (21.1; 32.3) | 30.4 (25.2; 39.9) |
| *Decelerate Lateral Lane Position Avg (m)* | -0.1 (-0.4; 0.1) | 0.2 (-0.2; 0.4) | 0.0 (-0.3; 0.3) |
| *Decelerate Lateral Lane Position Std (m)* | 0.1 (0.1; 0.2) | 0.2 (0.1; 0.2) | 0.1 (0.1; 0.2) |
| *Wait Duration (s)* | 4.4 (3.2; 5.2) | 4.2 (3.3; 5.6) | 6.3 (4.3; 10.1) |
| *Wait Distance (m)* | 3.2 (2.3; 4.4) | 5.2 (2.4; 6.9) | 7.8 (3.7; 13.1) |
| *Wait Lateral Lane Position Avg (m)* | -0.3 (-0.8; -0.2) | 0.2 (-0.2; 0.4) | 0.0 (-0.3; 0.4) |
| *Wait Lateral Lane Position Std (m)* | 0.1 (0.0; 0.1) | 0.1 (0.0; 0.2) | 0.1 (0.1; 0.3) |
| *Execute Duration (s)* | 2.4 (2.2; 2.8) | 2.6 (2.3; 3.2) | 3.2 (2.6; 3.9) |
| *Execute Distance (m)* | 10.8 (10.3; 11.7) | 11.6 (10.1; 13.6) | 11.9 (10.1; 13.2) |
| *Execute Lateral Lane Position Avg (m)* | -0.2 (-0.7; 0.0) | -0.1 (-0.3; 0.1) | -0.1 (-0.4; 0.2) |
| *Execute Lateral Lane Position Std (m)* | 0.5 (0.4; 0.7) | 0.4 (0.3; 0.5) | 0.5 (0.3; 0.6) |
| *Adjust Duration (s)* | 3.7 (3.4; 4.4) | 3.6 (3.4; 4.1) | 4.8 (4.1; 5.6) |
| *Adjust Lateral Lane Position Avg (m)* | 0.3 (-0.1; 0.8) | 0.7 (0.3; 1.0) | 0.7 (0.2; 1.0) |
| *Adjust Lateral Lane Position Std (m)* | 0.6 (0.4; 0.7) | 0.6 (0.4; 0.6) | 0.5 (0.4; 0.7) |
| *Stop Position (m)* | 6.1 (4.2; 7.2) | 8.0 (5.1; 10.6) | 9.3 (7.2; 11.4) |

**Table 3**: *Tabulated list of subtask metrics (duration, distance, lateral lane position average, and lateral lane position standard deviation) across the three age-groups for all driving tasks, quoted as median values and interquartile ranges. False discovery rate corrected significant differences (q < 0.05) are highlighted in grey. Avg = average; Std = standard deviation.*

|  | **Young** | **Middle-Aged** | **Old** |
| --- | --- | --- | --- |
|  | **Driving Task L** | | |
| *Overall Duration (s)* | 0.13 | -0.04 | 0.18 |
| *Lateral Lane Position Avg (m)* | 0.17 | -0.31 | 0.17 |
| *Lateral Lane Position Std (m)* | -0.01 | 0.24 | 0.25 |
|  | **Driving Task LT** | | |
| *Overall Duration (s)* | -0.15 | 0.34 | 0.18 |
| *Lateral Lane Position Avg (m)* | 0.28 | 0.26 | 0.27 |
| *Lateral Lane Position Std (m)* | 0.01 | -0.23 | -0.02 |
|  | **Driving Task LTA** | | |
| *Overall Duration (s)* | -0.04 | 0.56 | 0.25 |
| *Lateral Lane Position Avg (m)* | 0.33 | 0.36 | 0.30 |
| *Lateral Lane Position Std (m)* | 0.12 | -0.38 | -0.09 |
|  | **Driving Task R** | | |
| *Overall Duration (s)* | 0.07 | 0.29 | 0.17 |
| *Lateral Lane Position Avg (m)* | 0.20 | -0.33 | 0.22 |
| *Lateral Lane Position Std (m)* | -0.18 | 0.05 | -0.16 |
|  | **Driving Task RP** | | |
| *Overall Duration (s)* | -0.05 | 0.15 | 0.18 |
| *Lateral Lane Position Avg (m)* | 0.03 | 0.03 | 0.14 |
| *Lateral Lane Position Std (m)* | -0.44 | 0.13 | -0.14 |

**Table 4**: *Tabulated list of Spearman correlation coefficients with age for all total task metrics (overall duration, lateral lane position average, and lateral lane position standard deviation) across the three age-groups for all intersection-based driving tasks, quoted as median values and interquartile ranges. False discovery rate corrected significant correlations with age (q < 0.05) are highlighted in grey. Avg = average; Std = standard deviation.*

|  | **Young** | **Middle-Aged** | **Old** |
| --- | --- | --- | --- |
|  | **Driving Task S** | | |
| *Overall Duration (s)* | -0.00 | 0.13 | 0.07 |
| *Lateral Lane Position Avg (m)* | 0.44 | -0.08 | 0.44 |
| *Lateral Lane Position Std (m)* | -0.36 | 0.12 | 0.43 |
|  | **Driving Task SA** | | |
| *Overall Duration (s)* | -0.01 | 0.17 | 0.10 |
| *Lateral Lane Position Avg (m)* | 0.40 | 0.07 | 0.35 |
| *Lateral Lane Position Std (m)* | -0.47 | 0.19 | 0.46 |
|  | **Driving Task L** | | |
| *Approach Duration (s)* | -0.06 | 0.33 | -0.22 |
| *Approach Distance (m)* | -0.10 | 0.35 | -0.27 |
| *Approach Lateral Lane Position Avg (m)* | 0.23 | 0.02 | 0.44 |
| *Approach Lateral Lane Position Std (m)* | -0.01 | 0.33 | 0.30 |
| *Decelerate Duration (s)* | 0.02 | -0.30 | 0.21 |
| *Decelerate Distance (m)* | 0.02 | -0.23 | 0.22 |
| *Decelerate Lateral Lane Position Avg (m)* | 0.23 | -0.14 | 0.40 |
| *Decelerate Lateral Lane Position Std (m)* | -0.08 | 0.05 | 0.40 |
| *Wait Duration (s)* | 0.08 | 0.05 | 0.12 |
| *Wait Distance (m)* | -0.08 | 0.10 | 0.25 |
| *Wait Lateral Lane Position Avg (m)* | 0.28 | -0.18 | 0.23 |
| *Wait Lateral Lane Position Std (m)* | -0.52 | 0.27 | 0.29 |
| *Execute Duration (s)* | 0.30 | -0.10 | 0.07 |
| *Execute Distance (m)* | 0.31 | -0.32 | 0.15 |
| *Execute Lateral Lane Position Avg (m)* | 0.35 | -0.20 | 0.47 |
| *Execute Lateral Lane Position Std (m)* | -0.03 | -0.16 | 0.12 |
| *Adjust Duration (s)* | -0.01 | 0.10 | 0.22 |
| *Adjust Lateral Lane Position Avg (m)* | 0.12 | -0.05 | 0.34 |
| *Adjust Lateral Lane Position Std (m)* | -0.28 | -0.41 | -0.03 |
| *Stop Position (m)* | 0.31 | -0.37 | 0.23 |
|  | **Driving Task LT** | | |
| *Approach Duration (s)* | 0.11 | 0.12 | -0.17 |
| *Approach Distance (m)* | 0.11 | 0.05 | -0.20 |
| *Approach Lateral Lane Position Avg (m)* | 0.23 | 0.05 | 0.34 |
| *Approach Lateral Lane Position Std (m)* | 0.14 | 0.26 | 0.19 |
| *Decelerate Duration (s)* | -0.03 | 0.10 | 0.00 |
| *Decelerate Distance (m)* | -0.13 | -0.10 | 0.02 |
| *Decelerate Lateral Lane Position Avg (m)* | 0.32 | 0.10 | 0.39 |
| *Decelerate Lateral Lane Position Std (m)* | 0.11 | 0.26 | 0.18 |
| *Wait Duration (s)* | -0.08 | 0.33 | 0.22 |
| *Wait Distance (m)* | -0.27 | 0.15 | 0.34 |
| *Wait Lateral Lane Position Avg (m)* | 0.22 | 0.05 | 0.34 |
| *Wait Lateral Lane Position Std (m)* | -0.12 | 0.16 | 0.38 |
| *Execute Duration (s)* | -0.19 | 0.00 | 0.06 |
| *Execute Distance (m)* | 0.10 | -0.19 | 0.20 |
| *Execute Lateral Lane Position Avg (m)* | 0.31 | -0.07 | 0.50 |
| *Execute Lateral Lane Position Std (m)* | 0.03 | 0.14 | 0.39 |
| *Adjust Duration (s)* | -0.38 | 0.25 | 0.01 |
| *Adjust Lateral Lane Position Avg (m)* | 0.28 | -0.22 | 0.56 |
| *Adjust Lateral Lane Position Std (m)* | -0.20 | 0.03 | 0.12 |
| *Stop Position (m)* | -0.00 | 0.00 | 0.27 |
|  | **Driving Task LTA** | | |
| *Approach Duration (s)* | 0.12 | 0.09 | -0.24 |
| *Approach Distance (m)* | -0.03 | 0.13 | -0.20 |
| *Approach Lateral Lane Position Avg (m)* | 0.58 | -0.03 | 0.36 |
| *Approach Lateral Lane Position Std (m)* | -0.21 | 0.31 | 0.17 |
| *Decelerate Duration (s)* | -0.02 | 0.09 | -0.05 |
| *Decelerate Distance (m)* | -0.06 | -0.02 | 0.04 |
| *Decelerate Lateral Lane Position Avg (m)* | 0.46 | -0.04 | 0.38 |
| *Decelerate Lateral Lane Position Std (m)* | -0.08 | -0.01 | 0.14 |
| *Wait Duration (s)* | -0.06 | 0.54 | 0.27 |
| *Wait Distance (m)* | -0.03 | 0.10 | 0.43 |
| *Wait Lateral Lane Position Avg (m)* | 0.47 | 0.08 | 0.30 |
| *Wait Lateral Lane Position Std (m)* | -0.21 | -0.00 | 0.43 |
| *Execute Duration (s)* | 0.15 | -0.15 | -0.07 |
| *Execute Distance (m)* | 0.27 | -0.37 | 0.08 |
| *Execute Lateral Lane Position Avg (m)* | 0.57 | 0.00 | 0.49 |
| *Execute Lateral Lane Position Std (m)* | -0.06 | 0.05 | 0.27 |
| *Adjust Duration (s)* | -0.17 | 0.21 | 0.08 |
| *Adjust Lateral Lane Position Avg (m)* | 0.46 | -0.14 | 0.55 |
| *Adjust Lateral Lane Position Std (m)* | -0.45 | -0.03 | 0.03 |
| *Stop Position (m)* | 0.13 | -0.25 | 0.21 |
|  | **Driving Task R** | | |
| *Approach Duration (s)* | 0.11 | 0.20 | -0.22 |
| *Approach Distance (m)* | 0.06 | 0.05 | -0.25 |
| *Approach Lateral Lane Position Avg (m)* | -0.42 | 0.25 | -0.31 |
| *Approach Lateral Lane Position Std (m)* | -0.09 | 0.24 | 0.22 |
| *Decelerate Duration (s)* | -0.07 | 0.05 | 0.06 |
| *Decelerate Distance (m)* | -0.08 | -0.05 | 0.06 |
| *Decelerate Lateral Lane Position Avg (m)* | -0.35 | 0.42 | -0.39 |
| *Decelerate Lateral Lane Position Std (m)* | -0.43 | 0.14 | 0.25 |
| *Wait Duration (s)* | 0.09 | 0.25 | 0.28 |
| *Wait Distance (m)* | -0.03 | 0.17 | 0.32 |
| *Wait Lateral Lane Position Avg (m)* | -0.12 | 0.31 | -0.37 |
| *Wait Lateral Lane Position Std (m)* | 0.03 | 0.31 | 0.33 |
| *Execute Duration (s)* | -0.06 | 0.06 | -0.21 |
| *Execute Distance (m)* | -0.23 | -0.19 | -0.07 |
| *Execute Lateral Lane Position Avg (m)* | -0.46 | 0.19 | -0.26 |
| *Execute Lateral Lane Position Std (m)* | -0.07 | 0.13 | 0.19 |
| *Adjust Duration (s)* | -0.04 | 0.18 | 0.09 |
| *Adjust Lateral Lane Position Avg (m)* | -0.48 | -0.19 | 0.11 |
| *Adjust Lateral Lane Position Std (m)* | -0.24 | -0.17 | 0.13 |
| *Stop Position (m)* | -0.20 | -0.02 | 0.24 |
|  | **Driving Task RP** | | |
| *Approach Duration (s)* | 0.06 | 0.25 | -0.06 |
| *Approach Distance (m)* | -0.05 | 0.29 | -0.09 |
| *Approach Lateral Lane Position Avg (m)* | -0.35 | 0.09 | -0.62 |
| *Approach Lateral Lane Position Std (m)* | -0.27 | 0.15 | 0.31 |
| *Decelerate Duration (s)* | -0.02 | -0.14 | -0.01 |
| *Decelerate Distance (m)* | -0.02 | -0.31 | -0.06 |
| *Decelerate Lateral Lane Position Avg (m)* | -0.23 | 0.10 | -0.37 |
| *Decelerate Lateral Lane Position Std (m)* | -0.35 | -0.00 | 0.24 |
| *Wait Duration (s)* | 0.09 | 0.09 | 0.26 |
| *Wait Distance (m)* | -0.19 | 0.20 | 0.30 |
| *Wait Lateral Lane Position Avg (m)* | 0.03 | 0.32 | -0.11 |
| *Wait Lateral Lane Position Std (m)* | -0.38 | 0.13 | 0.14 |
| *Execute Duration (s)* | 0.00 | -0.08 | -0.13 |
| *Execute Distance (m)* | 0.08 | -0.12 | -0.03 |
| *Execute Lateral Lane Position Avg (m)* | -0.26 | -0.06 | -0.43 |
| *Execute Lateral Lane Position Std (m)* | 0.04 | 0.11 | 0.22 |
| *Adjust Duration (s)* | -0.16 | 0.13 | 0.07 |
| *Adjust Lateral Lane Position Avg (m)* | -0.26 | -0.24 | 0.17 |
| *Adjust Lateral Lane Position Std (m)* | -0.43 | -0.03 | 0.10 |
| *Stop Position (m)* | 0.04 | -0.07 | 0.23 |

**Table 5**: *Tabulated list of Spearman correlation coefficients with age for all subtask metrics (duration, distance, lateral lane position average, and lateral lane position standard deviation) for all intersection-based driving tasks as well as for all total task metrics (overall duration, lateral lane position average, and lateral lane position standard deviation) for straight driving tasks across the three age-groups, quoted as median values and interquartile ranges. False discovery rate corrected significant correlations with age (q < 0.05) are highlighted in grey. Avg = average; Std = standard deviation.*
